# VISTA expressed in tumour cells regulates T cell function

**DOI:** 10.1038/s41416-018-0313-5

**Published:** 2018-11-09

**Authors:** Kumuluzi Mulati, Junzo Hamanishi, Noriomi Matsumura, Kenji Chamoto, Nathan Mise, Kaoru Abiko, Tsukasa Baba, Ken Yamaguchi, Naoki Horikawa, Ryusuke Murakami, Mana Taki, Kharma Budiman, Xiang Zeng, Yuko Hosoe, Miyuki Azuma, Ikuo Konishi, Masaki Mandai

**Affiliations:** 1 Department of Gynecology and Obstetrics, Kyoto University Graduate School of Medicine, Kyoto, Japan; 2 Department of Obstetrics and Gynecology, Kindai University, Osaka, Japan; 3 Department of Immunology and Genomic Medicine, Graduate School of Medicine, Kyoto University, Kyoto, Japan; 4 Department of Environmental Preventive Medicine, Jichi Medical University, Tochigi, Japan; 5 Department of Obstetrics and Gynecology, Kyoto Medical Center, Kyoto, Japan; 6 Department of Molecular Immunology, Tokyo Medical and Dental University, Tokyo, Japan

**Keywords:** Tumour immunology, Gynaecological cancer

## Abstract

**Background:**

V-domain Ig suppressor of T cell activation (VISTA) is a novel inhibitory immune-checkpoint protein. VISTA expression on tumour cells and the associated regulatory mechanisms remain unclear. We investigated VISTA expression and function in tumour cells, and evaluated its mechanism and activity.

**Methods:**

VISTA in tumour cells was assessed by tissue microarray analysis, immunohistochemical staining and western blot. A series of in vitro assays were used to determine the function of tumour-expressed VISTA. In vivo efficacy was evaluated in syngeneic models.

**Results:**

VISTA was highly expressed in human ovarian and endometrial cancers. Upregulation of VISTA in endometrial cancer was related to the methylation status of the VISTA promoter. VISTA in tumour cells suppressed T cell proliferation and cytokine production in vitro, and decreased the tumour-infiltrating CD8+ T cells in vivo. Anti-VISTA antibody prolonged the survival of tumour-bearing mice.

**Conclusions:**

This is the first demonstration that VISTA is highly expressed in human ovarian and endometrial cancer cells, and that anti-VISTA antibody treatment significantly prolongs the survival of mice bearing tumours expressing high levels of VISTA. The data suggest that VISTA is a novel immunosuppressive factor within the tumour microenvironment, as well as a new target for cancer immunotherapy.

## Background

Tumour-induced immune suppression is a major obstacle for cancer immunotherapies that seek to eliminate cancer cells. Various mechanisms contribute to this immune activity. Recently, the targeting of B7-family regulatory molecules was demonstrated to be a promising approach for the treatment of immune-related diseases, such as autoimmunity and cancer.^1,2^ This is illustrated by the ability of blocking monoclonal antibodies against cytotoxic T-lymphocyte-associated protein 4 (CTLA-4), programmed cell death protein 1 (PD-1), and programmed death-ligand 1 (PD-L1) to enhance antitumour immunity in several solid tumours,^3–6^ including gynaecologic cancers.^7–9^

Ovarian cancer is the most lethal gynaecologic cancer worldwide, and the fourth most common cause of cancer-related death in women. More than 75% of patients are diagnosed at an advanced stage.^10,11^ Although chemotherapy is effective in the majority of ovarian cancer cases, more than 70% of patients suffer from recurrence and eventually develop chemoresistance.^12^ Endometrial cancer is another of the most common gynaecological cancers. The prognosis of low-risk endometrial cancer is generally favourable. However, for high-risk patients, chemotherapeutic options are limited, and no molecular therapies have been approved. Therefore, the development of novel therapeutic strategies, including immunotherapy, is urgently required.^13,14^

Although PD-1 blockade therapy has been approved for several types of cancers, approximately half of the patients treated with immune-checkpoint inhibitors do not benefit from these therapies,^15^ and the clinical responses to PD-1 signal inhibitors, such as anti-PD-1 or PD-L1 antibodies, have been unsatisfactory for both ovarian (11.5–15% overall) and endometrial cancers (13% overall).^7,16–18^ Therefore, combinatorial treatments, using conventional chemotherapy and new treatment strategies, must be developed to successfully treat cancers that are refractory or resistant to anti-PD-1 therapy. However, some combinatorial treatments involving immunotherapy are associated with severe immune-related adverse events.^15^ Therefore, the identification of new checkpoint molecules and elucidation of their expression and function in tumours would be of tremendous value in designing effective new anti-cancer therapies.

V-domain Ig suppressor of T cell activation (VISTA), which is also known as C10orf54, differentiation of ESC-1 (Dies1), DD1α, platelet receptor Gi24 precursor, and PD-1 homolog (PD-1H), is a member of the B7-family that shares significant homology with PD-L1 and PD-L2.^19–23^ Human and murine VISTA proteins are predominantly expressed on haematopoietic cells and myeloid cells, as well as being weakly expressed on T cells.^24^ Although VISTA is present in non-cancer cells at tumour sites, its expression in tumour cells, including gynaecologic malignancies, has not yet been examined. In this study, we examined the expression of VISTA in tumour cells, explored the mechanisms underlying the expression and immunosuppressive functions of VISTA in these cells, and examined the influence of blocking VISTA in VISTA-positive tumour cells on survival. The findings implicate VISTA as a novel potential target for cancer immunotherapy.

## Materials and methods

### VISTA gene expression microarray analysis

*VISTA* mRNA expression in 30 types of malignant tumours was determined from The Cancer Genome Atlas (TCGA) dataset using cBioportal (http://www.cbioportal.org/index.do)^25^ and in the Cancer Cell Line Encyclopedia (CCLE; http://www.broadinstitute.org/ccle), which contains data from 52 ovarian cancer cell lines and 27 endometrial cancer cell lines. The publicly available gene expression microarray dataset Gene Set Enrichment (GSE) 17025^26^ was used to measure VISTA expression in normal endometrium tissue or endometrial cancer tissue.

### Patients and samples

For immunohistochemical analysis of VISTA, formalin-fixed, paraffin-embedded specimens were obtained from 92 patients diagnosed with ovarian cancer who underwent primary operations and from 82 patients with endometrial cancer treated at the Department of Obstetrics and Gynaecologic of Kyoto University from 1996 to 2012. Relevant clinical data were collected by retrospective review of the patients’ files.

### Immunohistochemical (IHC) staining and immunofluorescence

IHC staining was performed as previously described^27,28^ using anti-VISTA polyclonal antibody (Atlas Antibodies HPA007968, lot: A89595) or monoclonal antibodies (AMAb91252, lot: 03077; AMAb91253, lot: 03078; Atlas Antibodies, Bromma, Sweden). Two independent gynaecological pathologists blinded to the clinical data examined the stained sections. Staining scores were calculated based on the degree of staining (−, 0; +, 1; ++, 2; +++, 3) and percentage of area stained. Staining scores were calculated as percentage ×0 (degree −) + percentage ×1 (degree +) + percentage ×2 (degree ++) + percentage ×3 (degree +++).

Immunofluorescence of VISTA on CD8+ T cells involved double staining with antibodies to VISTA (AMAb91252, lot: 03077; Atlas Antibodies) and CD8 (clone: C8/144B; DAKO, Carpinteria, CA, USA) using TSA Plus fluorescein evaluation kit (PerkinElmer, Waltham, MA, USA).

### Cell culture and transfection

The ID8 mouse ovarian cancer cell line^29^ was kindly provided by Dr. Katherine Roby (The University of Kansas Medical Center, Kansas City, KS, USA). ID8 cells were cultured and maintained in RPMI 1640 medium (Invitrogen, Carlsbad, CA, USA) supplemented with 10% (v/v) heat-inactivated foetal bovine serum (FBS; Biowest, San Marcos, TX, USA) and penicillin–streptomycin (100 IU/ml penicillin and 100 μg/ml streptomycin; Nacalai Tesque, Kyoto, Japan) in an atmosphere containing 5% CO_2_ at 37 °C. The OV2944-HM-1 (HM-1) mouse ovarian cancer cell line was purchased from RIKEN BioResource Center (RIKEN BRC; Tsukuba, Japan) in January 2003 and cultured as described previously.^28^ All cell lines were regularly tested for mycoplasma contamination.

The VISTA-overexpressing cell lines ID8-VISTA and HM-1-VISTA were generated by retroviral transfection of pMXs-internal ribosome entry site-green fluorescent protein (pMXs-IRES-GFP) vector containing mouse VISTA cDNA. The full cDNA sequence was purchased from TaKaRa Bio (Shiga, Japan) and amplified by PCR using the AAACACGATAATACCCACCATGGGTGTCCC forward primer and TTATTTTATCGTCGACTTAGATGGCTTCAGA reverse primer. The expression vector was generated using the In-fusion HD Cloning Kit (TaKaRa Bio).

Human endometrial cancer cell lines JHUEM1, JHUEM-2, JHUEM7, and Ishikawa were purchased from RIKEN BRC; the AN3CA, HEC1A, KLE, RL95-2, and TEN cell lines were from ATCC (Manassas, VA, USA); and the HEC50B, HEC108, and SNG-M cell lines were from the Japanese Cell Resources Bank (JCRB; Tokyo, Japan). The COV504 human ovarian cancer cell line was purchased from the European Collection of Authenticated Cell Cultures (Porton Down, UK). The JHOM1, MCAS, OVKATE, SKOV3, A2780, HEYA8, JHOC5, and TOV112D cell lines were kindly provided by Dr. Susan K. Murphy from the Department of Obstetrics and Gynecology, Duke University (Durham, NC, USA). Immortalised human endometrial epithelial cells (EM cells) were kindly provided by Professor Satoru Kyo from the Department of Obstetrics and Gynecology, Shimane University, Japan and were maintained as previously described (PMID: 14633600). Cells were maintained in RPMI 1640 medium (Nikken, Irvine, CA, USA), or Dulbecco’s modified Eagle’s medium (DMEM)/Ham’s F12 (Invitrogen) supplemented with 10% (v/v) heat-inactivated FBS (Biowest) and penicillin–streptomycin (100 IU/ml penicillin and 100 μg/ml streptomycin; Nacalai Tesque) and were regularly tested for mycoplasma contamination. JHUEM1, JHUEM7, HEC1A, and COV504 cells used for further functional assays were authenticated by short tandem repeat analysis.

VISTA-knockdown human cell lines COV504-sh-VISTA and HEC1A-sh-VISTA were generated by lentiviral transfection of short hairpin RNAs (shRNAs) targeting VISTA using the human GIPZ c10orf54 shRNA viral particle set (clone ID V2LHS_263617, 315659, and 315662, gene target sequence 5′-CGAAACGGGAAGTACATAT-3′, 5′-GGGCACGATGTGACCTTCT-3′, and 5′-CGATGTGACCTTCTACAAG-3′; GE Healthcare, Buckinghamshire, UK). Control cell lines COV504-sh-Control and HEC1A-sh-Control were generated by transfection of a non-silencing control shRNA (GE Healthcare).

### Cell treatments

Recombinant mouse interferon-gamma (IFN-γ), interleukin (IL)-2, IL-4, IL-6, IL-17, or tumour necrosis factor-alpha (TNF-α; all from PeproTech, Montreal, Quebec, Canada) was added to the culture medium at a concentration of 20 ng/ml. The cultures were incubated for 24 h, after which the cells were analysed for VISTA expression. Human cell lines were treated with 20 ng/ml recombinant human IFN-γ (R&D Systems, Madison, WI, USA) or 5 ng/ml TGF-β (PeproTech) for 24 h before analysis.

### Cell proliferation assay

Established VISTA-overexpressing cells were seeded in 96-well plates (Asahi Glass, Tokyo, Japan) and incubated for 3 days. The number of viable cells in each well was examined every 24 h using the WST-1 assay (TaKaRa Bio).

### Western blotting

Patient samples used for western blot were prepared by sorting tumour tissue with epithelial cellular adhesion molecule (EpCAM) microbeads (Miltenyi Biotec, Bergisch, Germany). Cell lines were harvested and lysed in radio-immunoprecipitation assay (RIPA) buffer with protease inhibitor cocktail (EMD Biosciences, Billerica, MA, USA) and phosphatase inhibitor cocktail (Nacalai Tesque). Anti-human VISTA antibody (1:1000 dilution, ab201565; Abcam, Cambridge, UK) and anti-glyceraldehyde-3-phosphate dehydrogenase (GAPDH) antibody (1:1000 dilution, ab9484; Abcam) were used for western blotting.

### Reverse transcription PCR and real-time quantitative PCR

Total RNA was extracted from cell lines or frozen tissues using the RNeasy Mini Kit (Qiagen, Valencia, CA, USA). The Transcriptor High-Fidelity cDNA Synthesis Kit (Roche Diagnostics, Risch-Rotkreuz, Switzerland) was used for cDNA synthesis. For reverse transcription (RT)-PCR, cDNAs were amplified using a thermal cycler. Real-time quantitative PCR was carried out by amplification of the target genes, using *GAPDH* mRNA as a reference, on a Light Cycler 480-II (Roche Diagnostics). Primers were as follows: *Gapdh*, forward: 5′-gggttcctataaatacggactgc-3′, reverse: 5′-ccattttgtctacgggacga-3′; *Vista*, forward: 5′-gacaggtggcctctcacc-3′, reverse: 5′-ttttcgattccttgggtgtt-3′; *GAPDH* forward: 5′-agccacatcgctcagacac-3′, reverse: 5′-gcccaatacgaccaaatcc-3′; *VISTA* forward: 5′-atccctgctcttcgctctct-3′, reverse: 5′-cctcgggacagacatacagg-3′. Relative expression levels were calculated using the 2^−^^∆∆Ct^ method.

### Flow cytometry

Cultured mouse cancer cells were incubated with allophycocyanin-conjugated anti-VISTA antibody (clone MH5A, BD BioLegend, San Diego, CA, USA) or a matched isotype control (BioLegend). Alexa647-conjugated anti-human VISTA monoclonal antibody (mAb) (MIH65.rMAB; BD Biosciences, San Jose, CA, USA). Spleens and tumours were collected from tumour-bearing mice. Cells were stained with the following antibodies: anti-mouse CD3e (PerCP, clone: 145-2c11; BD Biosciences), anti-mouse CD45 (APC-cy7, clone: 30-F11; BioLegend), anti-mouse CD8a (PE-Vio770, clone: 53-6.7; Miltenyi Biotics), anti-mouse CD4 (APC, clone: RM4-5; BD Pharmingen, San Jose, CA, USA); anti-mouse Gr-1 antibody (PE, clone: RB6-8C5;, BD Biosciences), and anti-mouse CD11b antibody (APC, clone: M1/70; TONBO Biosciences, San Diego, CA, USA) or matched isotype controls. A BD Cytofix/Cytoderms Fixation/ Permeabilization Kit (BD Biosciences) was used for intracellular staining of IFN-γ (phycoerythrin PE, clone: XMG1.2; BD Pharmingen). Nonviable cells were stained with 7-amino-actinomycin D or 4′,6-diamidino-2-phenylindole and gated out. Flow cytometry analysis was performed on a MACS Quant Analyzer 10 (Miltenyi Biotec). Data analysis was performed using the FlowJo software (Tree Star, Eugene, OR, USA).

### Cytotoxicity assay

As effectors, CD8+ T cells were purified from OT-1 mouse spleen cells by magnetic bead selection using the CD8a+ T cell Isolation Kit (Miltenyi Biotec), and then stimulated and expanded for 6–8 days in the presence of CD3/CD28 Dynabeads (Gibco, Franklin Lakes, NJ, USA) and 100 IU IL-2 (TONBO Biosciences). RPMI 1640 medium supplemented with 10% FBS, 50 μmol/l 2-mercaptoethanol (Nacalai Tesque), 2 mmol/l l-glutamine (Invitrogen), and penicillin–streptomycin (Nacalai Tesque) was used for lymphocyte cultures. As target cells, ID8-VISTA or ID8-control cells were treated with 20 ng/ml IFN-γ (Peproech) overnight to boost major histocompatibility complex 1 (MHC-I) expression, and then loaded with 10 μg/ml ovalbumin (OVA) peptide (MBL International, Woburn, MA, USA) at 37 °C for 1 h. Cells without OVA loading served as negative controls. Target cells were co-cultured with effector cells at various effector-to-target (E/T) ratios. After 4 h of incubation, the levels of lactate dehydrogenase in the co-culture supernatant were determined using the CytoTox 96 Non-Radioactive Cytotoxicity Kit (Promega). The percentage of lysis was calculated according to a modified standard formula: [(Optical density [OD] experimental − OD spontaneous targets − OD spontaneous effectors)/(OD maximum − OD spontaneous targets)] × 100.

### Carboxyfluorescein succinimidylester (CFSE) cell division assay

Human T cells were isolated from peripheral blood mononuclear cells (PBMCs) of healthy donors using the Human Pan-T Cell Isolation Kit (Miltenyi Biotec). T cells were stained with 5 µmol/l of 5-(−6)-carboxyfluorescein diacetate succinimidylester (CFSE; Molecular Probes, Eugene, OR, USA) for 10 min at 37 °C, and then washed three times with RPMI containing 10% FBS. The cells were co-cultured with mitomycin-treated HEC1A human endometrial cancer cells or COV504 human ovarian cancer cells, or their corresponding sh-VISTA cell lines, at a 1:1 ratio in the presence of CD3/CD28 Dynabeads (Gibco). Each condition was tested in triplicate. After 72 h, cells were collected, and T cell proliferation was examined by flow cytometry.

In mouse models, the same settings were used to analyse division of CSFE-labelled mouse T cells derived from C57BL/6N or B6C3F1 mice. Cells were isolated using the Mouse Pan-T Cell Isolation Kit (Miltenyi Biotec) and co-cultured with ID8 or HM-1 mouse ovarian cancer cells that did or did not express VISTA at various ratios. Nonviable cells were stained with 4′,6-diamidino-2-phenylindole and gated out. Anti-human CD3 (PE, clone: UCHT1, BD Pharmingen) and anti-mouse CD3 (APC-Cy7, clone: 17A2, TONBO Biosciences) were used for flow cytometry detection. Anti-mouse VISTA antibody (MIH63, rat IgG2a) was added at the concentration of 0.5, 1, 2.5, or 5 µg/ml at an E/T ratio of 1:1.

### Enzyme-linked immunosorbent assay (ELISA)

Human or mouse IFN-γ, IL-2, and TNFα ELISA kit (Qiagen, Valencia, CA, USA) were used to analyse cytokine production. All standards and samples were run in triplicate.

### Detection of microRNA (miRNA)

The miScript II RT Kit (Qiagen) was used to synthesise the first-strand cDNA, and miScript SYBR Green Kit (Qiagen) was used to detect miR-125a-5p or miR-506 expression. All samples were run in triplicate.

### 5-Aza-2′-deoxycytidine (5-aza-dC) treatment

JHUEM1 and JHUEM7 human endometrial cancer cell lines, the EM immortalised endometrial epithelial cell line, and the COV504, SKOV3, and A2780 human ovarian cancer cell lines were seeded in 6-well plates. 5-Aza-dC (Decitabine, ab120842; Abcam), a demethylating agent, was added to the cells at a concentration of 5 μM. Decitabine and medium were refreshed every day. Control cells received no drug treatment. Cells were harvested for total RNA extraction 3 days after treatment. VISTA expression was determined by real-time PCR.

### Epigenetic analysis

DNA methylation status of the *VISTA* promoter was analysed by bisulphite PCR sequencing of cloned alleles. Genomic DNA was purified with DNeasy Blood and Tissue Kit (Qiagen) from high or low VISTA-expressing endometrial/ovarian cancer cell lines, the EM immortalised endometrial epithelial cell line, and frozen sections of clinical samples. One microgram of DNA was bisulphite-converted using the EpiTect Fast DNA bisulphite Kit (Qiagen). For bisulphite sequencing analyses, primers were designed to recognise sodium bisulphite-converted DNA encompassing three regions within the human *VISTA* promoter region in the published human genome assembly (GRCh38/hg38). Region 1 was chr 10: 71, 773, 235– 71, 773, 735; region 2 was chr 10: 71, 773, 500–71, 773, 850; and region 3 was chr 10: 71, 774,000–71,774,300. Primers were designed using the Primer3plus software (http://www.bioinformatics.nl/cgi-bin/primer3plus/primer3plus.cgi). For amplification of region 2, PCR products were further amplified with a second round of nested PCR to obtain sufficient quantities of DNA. Primers used for region 2 were as follows: first round, forward: 5′-ttttagcgtcgtgttttaggtattc-3′, reverse: 5′-acctaccacataccaaaccctaatc-3′; second round, forward 5′-gtgtgggggaattgtttttt-3′, reverse: 5′-cccaaatcactcaaaaactaataactaa-3′. Temperature conditions for PCR were as follows: 98 °C for 3 min, 35 cycles of 98 °C for 10 s, 55 °C for 30 s, and 72 °C for 1 minute, followed by one cycle of 72 °C for 5 min. Using EpiTaqHS (for bisulfite-treated DNA, R110A: TaKaRa Bio), PCR products were purified using the QIAquick PCR Purification Kit (Qiagen) or QIAquick Gel Extraction Kit (Qiagen). The PCR products were then cloned into TA vectors (TA Cloning Kit, Invitrogen) and transfected into TOP10 *E. coli* (Thermo Fisher Scientific, Waltham, MA, USA). Plasmid DNA was extracted using the QIAprep Spin Miniprep Kit (Qiagen), and 8 to 15 clones of each specimen were subjected to standard sequencing analysis.

### Mice

Female C57BL/6 (B6) and B6C3F1 mice were purchased from CLEA Japan (Tokyo, Japan). OVA-specific T cell receptor transgenic mice (OT-1 mice) and CAG-GFP mice were purchased from The Jackson Laboratory (Bar Harbor, MN, USA) and interbred to generate OT-1-GFP mice. A total of 5 × 10^6^ ID8-VISTA or ID8-control cells, or 1 × 10^6^ HM-1-VISTA or HM-1 control cells, were separately intraperitoneally inoculated into B6 mice or B6C3F1 mice.

Hybridomas producing anti-VISTA mAb (MIH63, rat IgG2a) were generated as previously described.^30–32^ Anti-VISTA antibody treatment was initiated 3 days after tumour cell inoculation; antibody was administered intraperitoneally at 200 µg/mouse three times a week. Anti-PD-1 antibody provided by Ono Pharmaceutical (Osaka, Japan) was initiated on day 5 after tumour cell inoculation at a dose of 100 µg/mouse and administered once every 5 days. Rat IgG (200 μg/mouse) was used as a negative control.

### Statistical analyses

Results are shown as mean ± SEM as appropriate. Mann–Whitney *U* test was used for group comparisons in IHC analyses of human samples, microarray data, and ELISA results. Two-way ANOVA with Šidak’s multiple comparisons test was used for CTL assay analysis. The log-rank test was used for overall survival analysis. All statistical analyses were performed using the Prism 6 software (GraphPad Software, La Jolla, CA, USA). The level of significance was set as *p < 0.05, **p < 0.01, ***p < 0.001, and ****p < 0.0001.

## Results

### VISTA expression in endometrial and ovarian cancer cells

**Fig. 1:**
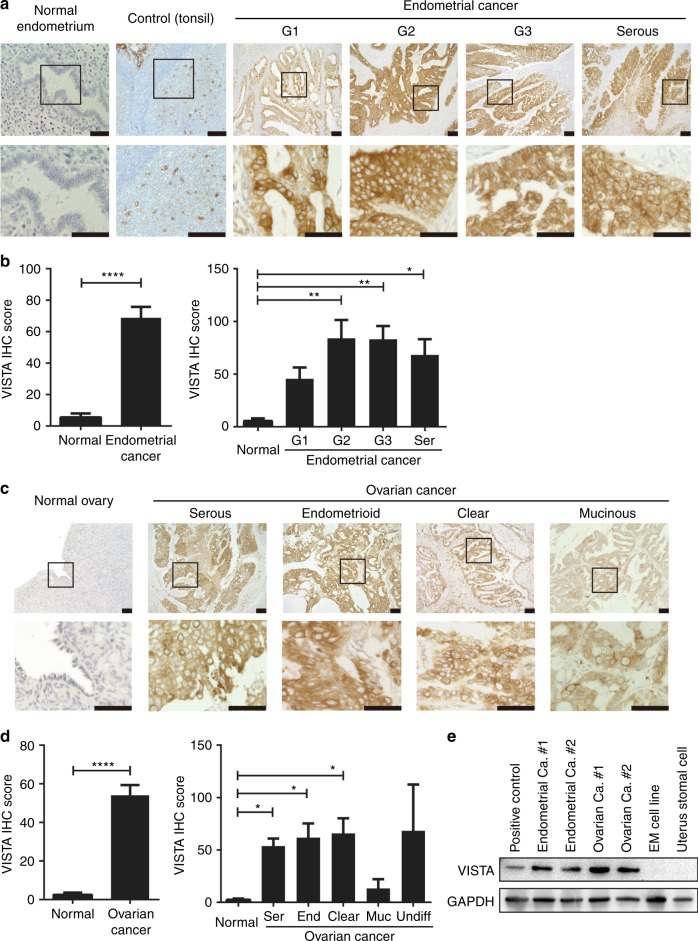
VISTA expression in malignant tumours. Immunohistochemistry (IHC) of formalin-fixed, paraffin-embedded tissues was used to evaluate VISTA expression in human endometrial cancer, ovarian cancer, or corresponding normal organs. **a** Representative VISTA staining of normal endometrium, positive-control tonsil tissue, G1–3, and serous endometrial cancer tissues. Scale bar denotes 100 μm. **b** VISTA IHC score in endometrial cancer. Data are shown as mean ± SEM. **c** VISTA staining of ovarian surface epithelium and serous, endometrioid, clear cell, and mucinous histological subtypes of ovarian cancer. Scale bar denotes 100 μm. **d** VISTA IHC score in ovarian cancer. Data are shown as mean ± SEM. Ser serous, End endometrioid, Muc mucinous, Undif undifferentiated. *p < 0.05, **p < 0.01, ***p < 0.001, ****p < 0.0001 by Mann–Whitney *U* test. **e** EpCAM + tumour cells were sorted from endometrial or ovarian cancer patient tumour tissues. VISTA expression was evaluated using western blot

Microarray data from TCGA revealed the high expression of *VISTA* mRNA in almost all of 30 of the malignant tumour types that were tested, including ovarian and endometrial cancers (Supplementary Figure 1A). Furthermore, IHC staining demonstrated that all 82 endometrial cancer specimens were positive for VISTA staining, which was detected both on the cell membrane and in the cytoplasm. By contrast, negative or low expression of VISTA was detected in 10 specimens of normal endometrial endometrium, including both proliferative and secretory phases. Tonsil tissue was used as a positive-control (Fig. 1a). The VISTA IHC score was significantly higher in endometrial cancer samples than in normal tissues. Moreover, it was higher in histopathologic grades G1, G2, and G3, and in serous types of endometrial cancer, than in normal endometrium (Fig. 1b). Gene expression of VISTA in the GSE17025 microarray dataset was consistent with the IHC data (Supplementary Figure 1B); higher expression was observed in stages I and II than in normal tissue (Supplementary Figure 1C). However, the Kaplan–Meier curve revealed no difference in survival of patients with endometrial cancer as a function of VISTA expression in the tumour (Supplementary Figure 1D). Patients were identified by number, and their characteristics are provided in Table 1.

**Table 1:** Pathological features of patients with endometrial cancer (*n* = 82)

Characteristic	VISTA expression (tumour)		*P*-value
	Low (47)	High (35)	
Age (mean ± SD)	58.34 ± 10.28	57.03 ± 10.57	0.574
FIGO stage			0.447
I	22	20	
II	1	3	
III	20	7	
IV	4	5	
Histological subtypes			0.376
Endometrioid G1	17	6	
Endometrioid G2	9	7	
Endometrioid G3	9	14	
Serous	13	7	
Myometrial invasion^a^			0.656
+	25/30.5%	21/25.6%	
−	21/25.6%	14/17.1%	
LVSI^a^			0.364
+	21/25.6%	20/24.4%	
−	24/29.3%	14/17.1%	
LN metastasis			0.639
+	16/19.5%	10/12.2%	
−	31/37.8%	25/30.5%	

Statistical significance was analysed by two-way ANOVA or *χ*^2^ test

*FIGO* International Federation of Gynecology and Obstetrics, *G* histological grade of endometrioid adenocarcinoma

^a^Data were missing in one case and three cases, respectively

VISTA expression was also observed in 84 of 92 (91.3%) ovarian cancer tissues of the following histopathologic subtypes: serous, endometrioid, clear cell, mucinous, and undifferentiated carcinoma. By contrast, VISTA expression was absent or low in normal ovarian epithelial specimens (Fig. 1c). The VISTA IHC score was significantly higher in ovarian cancer samples than in normal tissue, and showed higher distribution in the serous, endometrioid, and clear cell subtypes (Fig. 1d). No difference in VISTA expression was detected between primary and metastatic sites in 28 paired samples of ovarian cancers (Supplementary Figure 1E). VISTA was more highly expressed in stages I and II than in normal tissue (Supplementary Figure 1F). Furthermore, as with endometrial cancer result, there was no difference in survival as a function of VISTA expression (Supplementary Figure 1G). Pathological features of patients with ovarian cancer are provided in Table 2. VISTA status in tumour cells was confirmed using two monoclonal antibodies. Similar staining patterns were observed with all three types of antibodies (Supplementary Figure 1H), and mAb staining scores were significantly correlated with the polyclonal antibody staining scores (Supplementary Figure 1I).

**Table 2:** Pathological features of patients with ovarian cancer (*n* = 92)

Characteristic	VISTA expression (tumour)		*P*-value
	Low	High	
Age (mean ± SD)	52.98 ± 11.35	57.06 ± 10.51	0.086
FIGO stage			0.299
I	11	8	
II	5	4	
III	36	21	
IV	3	4	
Histological subtypes ^a^			0.314
Serous	33	20	
Endometrioid	8	7	
Clear cell	5	6	
Mucinous	3	0	
Undifferentiated	1	2	
Metastasis^a^			0.821
+	19/20.7%	13/14.1%	
−	37/40.2%	21/22.8%	

Statistical significance was analysed by two-way ANOVA or *χ*^2^ test

*FIGO* International Federation of Gynecology and Obstetrics

^a^Data were missing in seven and two cases, respectively

Immunofluorescence of four endometrial cancers upon the double staining for VISTA and CD8 showed that the samples of two patients were highly infiltrated with CD8+ T cells and highly correlated with VISTA expression, while the other two samples were less infiltrated with VISTA+ CD8+ T cells in the tumour site (Supplementary Figure 1J).

We also confirmed the IHC findings by western blotting. Tumour tissues from endometrial or ovarian cancer patients were homogenised and EpCAM+ tumour cells were sorted and tested for VISTA expression. The results of western blotting were consistent with the IHC staining data (Fig. 1e). Overall, these results demonstrate that VISTA is expressed in both endometrial and ovarian cancer cells.

### Silencing VISTA expression in human cancer cells restores T cell proliferation and cytokine secretion

**Fig. 2:**
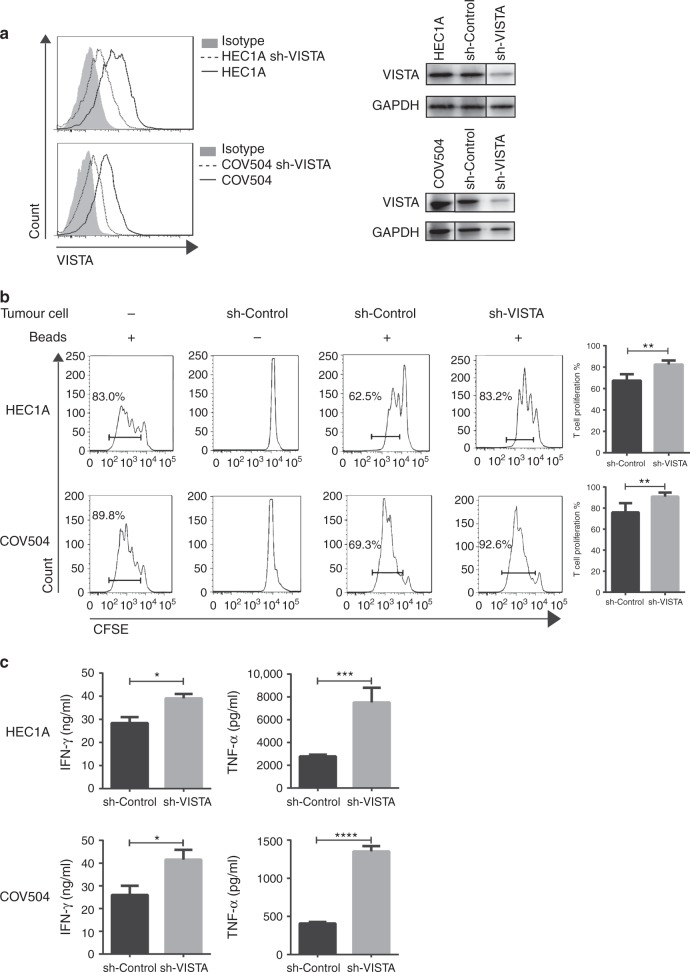
Silencing of VISTA expression in human cancer cells restores T cell proliferation and cytokine secretion. **a** Generation of sh-VISTA human endometrial cancer cell line (HEC1A) and sh-VISTA human ovarian cancer cell line (COV504). VISTA silencing was confirmed by flow cytometry (left) and western blotting (right). **b** T cells were purified from human PBMCs, labelled with CFSE, not stimulated or stimulated with CD3/CD28 beads, and co-cultured with mitomycin-treated sh-Control or sh-VISTA HEC1A or COV504 tumour cells at a 1:1 ratio. The percentage of proliferating T cells is indicated in each panel. ‘Beads’ indicates CD3/CD28 beads. The averaged results of four separate experiments are shown in the bar graphs at right. **c** Cytokine concentrations in the culture supernatant, determined by ELISA, are shown as mean ± SEM (*n* = 6). *p < 0.05, **p < 0.01, ***p < 0.001, ****p < 0.0001 by Mann–Whitney *U* test

We quantitatively confirmed VISTA gene expression in all 13 human endometrial cancer cell lines and nine human ovarian cancer cell lines (Supplementary Figure 2A, B). VISTA protein was analysed in nine human endometrial cancer cell lines and one immortalised endometrial epithelial cell (EM) line (Supplementary Figure 2C). Almost all these cancer cell lines endogenously expressed VISTA, whereas normal cell lines did not. Therefore, to assess the functional effect of VISTA expression, we established the HEC1A-sh-VISTA human endometrial cancer cell line and COV504-sh-VISTA human ovarian cancer cell line using three independent lentiviral shRNAs targeting VISTA. Silencing of VISTA expression was confirmed by RT-PCR, flow cytometry, and western blot based on control shRNA-transduced HEC1A (Fig. 2a, Supplementary Figure 2E). To evaluate the effect of VISTA expression in tumour cells, we co-cultured purified T cells from healthy donors with sh-VISTA or sh-control tumour cells, and measured T cell proliferation by flow cytometry. Tumour-free or bead-free samples were used as negative controls. Silencing of VISTA expression in HEC1A (HEC1A-sh-VISTA) restored the proliferation of T cells from 62.5 to 83.2%. Similarly, silencing of VISTA expression in COV504 (COV504-sh-VISTA) restored T cell proliferation from 69.3 to 92.6% (Fig. 2b, Supplementary Figure 3A, B). Moreover, both IFN-γ and TNFα were upregulated in the supernatants (Fig. 2c). These data indicate that endometrial and ovarian cancers express high levels of VISTA, and that silencing of VISTA expression in tumour cells can increase T cell proliferation and cytokine production.

### Methylation status correlates with VISTA expression in human endometrial cancer

VISTA shares significant homology with PD-L1, which is upregulated by IFN-γ. In addition, a connection between VISTA and TGF-β has been described.^33^ Hence, to elucidate the regulation of VISTA in cancer cells, we performed western blotting to assess the impact of IFN-γ and TGF-β on VISTA expression in several human ovarian or endometrial cancer cell lines, as well as an immortalised endometrial epithelial cell line. No significant effect was observed, with representative data presented in Supplementary Figure 2D. We then investigated the role of miRNAs on VISTA expression. Using TargetScan for human (http://www.targetscan.org/vert_71/), we predicted two miRNA candidates, miRNA125a and miRNA506, and assessed their expression levels in high or low VISTA-expressing human gynaecologic cancer cell lines. The expression of miRNA125A and miRNA506 did not correlate with VISTA expression (Supplementary Figure 4).

**Fig. 3:**
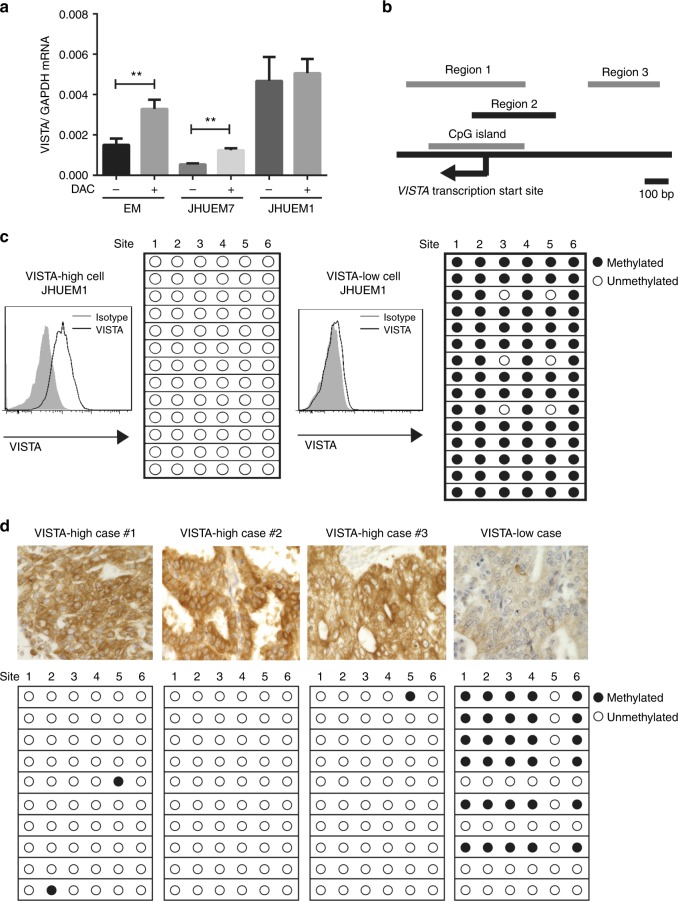
Methylation status correlates with the expression of VISTA in human endometrial cancer. **a** VISTA expression in endometrial cancer cell lines and immortalised endometrial epithelial cell line after decitabine treatment, as determined by RT-PCR. Data are presented as mean ± SEM (*n* = 6); **p < 0.01 by Mann–Whitney *U* test. **b** Positional relationship of the three selected promoter regions of *VISTA*. Scale bar denotes 100 bp. **c** Methylation status of promoter region 2 in endometrial cancer cell lines expressing high or low levels of VISTA. VISTA expression was confirmed using flow cytometry. **d** Methylation status of promoter region 2 in the clinical endometrial cancer specimens expressing high or low levels of VISTA. VISTA expression level was determined by IHC staining. Solid circle indicates methylated site and hollow circle indicates unmethylated site. Each column represents a CpG site, and each horizontal row represents a different sample clone

We next investigated whether VISTA expression was regulated by epigenetic events in tumour cell lines by evaluating changes in VISTA expression before and after a 3-day treatment with decitabine. The experiment was performed on two human endometrial cancer cell lines—JHUEM1 (high VISTA expression) and JHUEM7 (low VISTA expression)—as well as the EM immortalised endometrial epithelial cell line. RT-PCR analysis revealed that *VISTA* mRNA expression was significantly upregulated after decitabine treatment in JHUEM7 and EM cells (Fig. 3a), but not in JHUEM1 or human ovarian cancer cell cells (Supplementary Figure 5). Since decitabine is a recognised demethylating agent,^34^ we hypothesised that DNA methylation status influences VISTA expression in endometrial cancer. To explore this possibility, we analysed the methylation status of the *VISTA* promoter by bisulphite sequencing in JHUEM1 and JHUEM7 cells. The relative positions of three analysed regions upstream of the transcription start site of *VISTA* are shown in Fig. 3b. Bisulphite sequencing of region 2 revealed that the VISTA promoter was highly methylated in JHUEM7 cells (low VISTA expression), whereas in JHUEM1 cells (high VISTA expression) this region was free of methylation (Fig. 3c). Similar results were obtained in regions 1 and 3 (Supplementary Figure 6, 7). To confirm these results in patients, we selected several clinical specimens from our previous IHC analysis that expressed high or low levels of VISTA. The DNA methylation status of these clinical specimens was consistent with the results obtained in the cell lines (Fig. 3d). These findings suggested that VISTA expression in endometrial cancer is controlled by DNA methylation of its promoter.

### VISTA in tumour cells suppresses T cell proliferation and promotes evasion of cytotoxic T lymphocytes (CTLs)

**Fig. 4:**
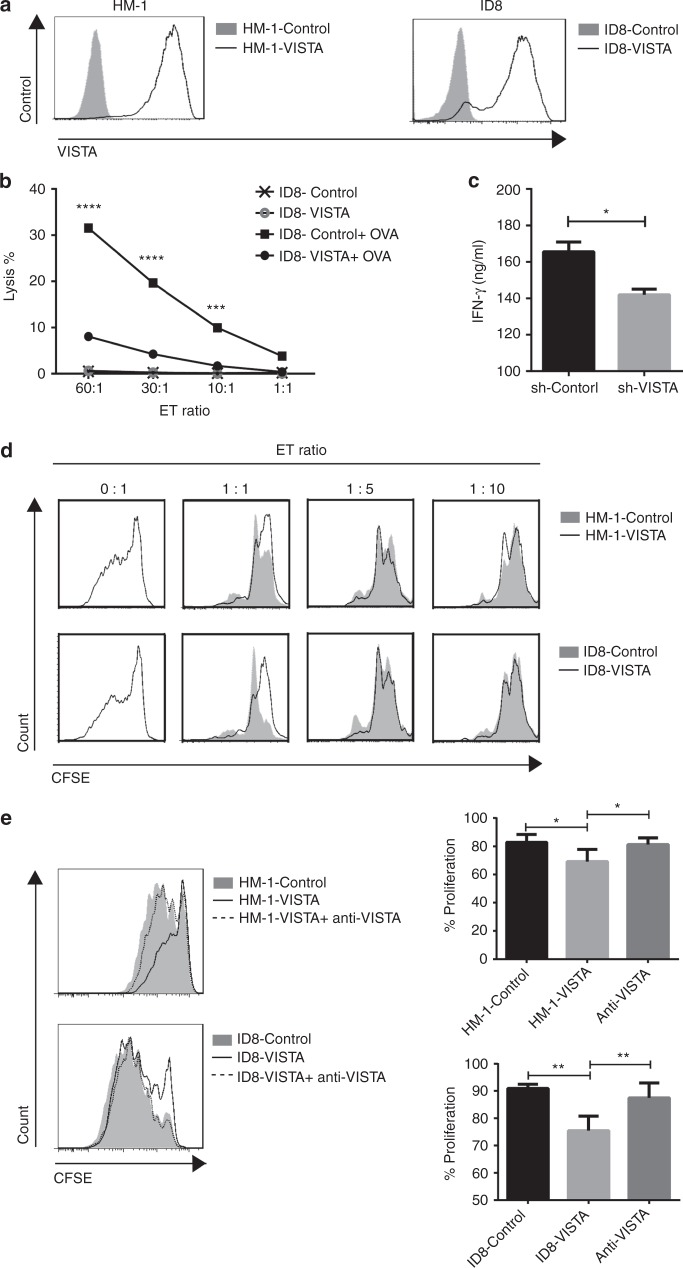
VISTA expressed in tumour cells suppresses T cell proliferation and protects tumour cells from CTLs. **a** Generation of VISTA-overexpressing mouse ovarian cancer cell lines (HM-1-VISTA and ID8-VISTA). VISTA expression was confirmed by flow cytometry. **b** CD8+ T cells from spleens of OT-1 mice were stimulated and expanded for 6–8 days with anti-CD3/CD28 beads and IL-2, and then co-cultured with OVA peptide–treated ID8-VISTA or ID8-control cells at various ratios. ID8 cells without OVA loading served as negative controls. Cytotoxicity assays were performed, and the data were analysed by two-way ANOVA with Šidak’s multiple comparisons test. **c** IFN-γ production in supernatant in **b** was measured by ELISA. Data are shown as mean ± SEM (*n* = 6); *p < 0.05 by Mann–Whitney *U* test. Data are representative of at least three separate experiments. **d** CD3+ T cells were purified from mouse spleen cells by magnetic bead selection, labelled with CFSE, and co-cultured for 3 days with HM-1-VISTA, ID8-VISTA, or control cells at various ratios in the presence of anti-CD3/CD28 beads. Data are representative of three separate experiments. **e** Representative histogram of T cell proliferation at the presence of 1 µg/ml anti-VISTA antibody (left) and percentage of T cell proliferation in the three groups (right). Data are representative of three separate experiments, presented as mean ± SEM (*n* = 3); *p < 0.05, **p < 0.01 by Mann–Whitney *U* test

HM-1 and ID8 mouse ovarian cancer cells expressed very low levels of VISTA, and the expression did not increase following treatment with IFN-γ, IL-4, IL-6, IL-10, IL-17, or TNF-α (Supplementary Figure 8A). Hence, we established HM-1-VISTA and ID8-VISTA VISTA-overexpressing mouse ovarian cancer cell lines by retroviral transfection (Fig. 4a). Proliferation assays revealed no differences in growth between VISTA-overexpressing and mock-transduced cells (Supplementary Figure 8B).

Next, to determine the function of VISTA in tumour cells, T cell cytotoxicity and proliferation assays were performed. When CD8+ T cells from OT-1 mice were co-incubated with ID8 cells with or without VISTA expression, higher levels of target cell lysis were observed in cultures with ID8-control cells than in those with ID8-VISTA cells, indicating that VISTA in tumour cells inhibits antigen-specific cytolysis of OT-1 CD8+ T cells (Fig. 4b). Measurements of cytokine levels in culture supernatants showed that VISTA in tumour cells significantly decreased the production of IFN-γ from OT-1 CD8+ T cells (Fig. 4c). Next, we co-cultured mitomycin-treated HM-1-VISTA or ID8-VISTA cell lines with T cells labelled with CFSE. HM-1-VISTA and ID8-VISTA suppressed T cell proliferation at an E/T ratio of 1:1 (Fig. 4d), although the immunosuppressive effect was too weak to be detected at a ratio of 1:5 or 1:10. This suppressive status of T cell proliferation upon co-cultured with HM-1-VISTA and ID8-VISTA was restored by anti-VISTA antibody treatment (Fig. 4e). These results indicate that VISTA in tumour cells inhibits T cell proliferation and protects cancer cells from antigen-specific cytolysis by CTLs, thereby contributing to immune evasion by tumour cells.

### VISTA in tumour cells is associated with T cell infiltration at the tumour site and impairs antitumour immunity

**Fig. 5:**
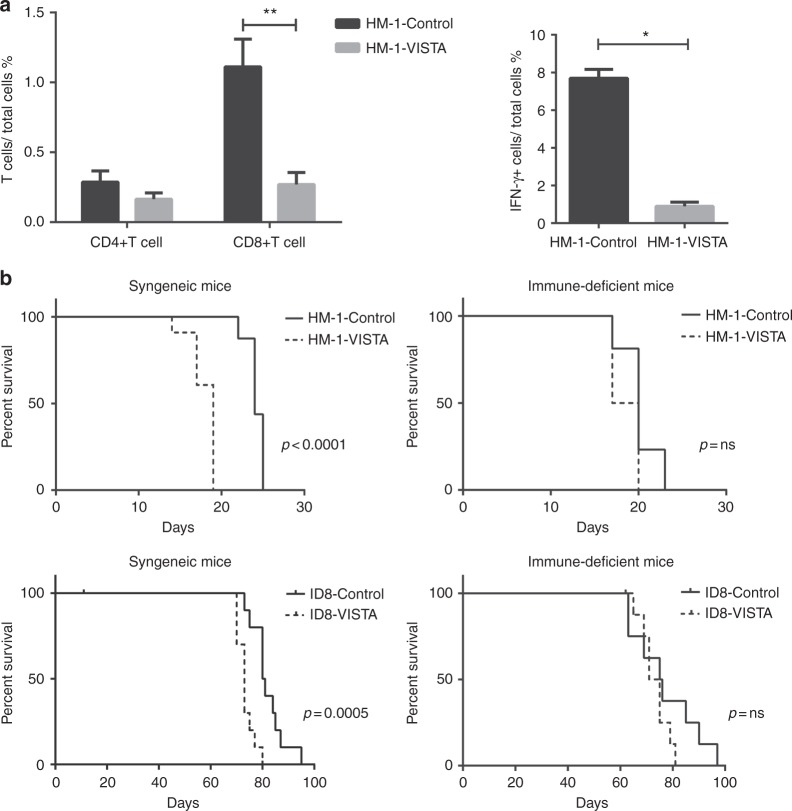
VISTA affects T cell infiltration in the tumour site and impairs antitumour immunity. **a** Percentages of T cells (left) and IFN-γ+ cells (right) relative to total cells in peritoneal tumours harvested from mice intraperitoneally injected with HM-1-VISTA or HM-1-control. Data are shown as mean ± SEM (*n* = 6); *p < 0.05, **p < 0.01 by Mann–Whitney *U* test. **b** Survival curves for syngeneic mice intraperitoneally inoculated with HM-1-control or HM-1-VISTA (upper left) (*p* < 0.0001, *n* = 6) and for immune-deficient mice (upper right) (*p* = non-significant, *n* = 6). Survival curves for syngeneic mice intraperitoneally inoculated with ID8-control and ID8-VISTA (bottom left) (*p* = 0.0005, *n* = 12); and for immune-deficient mice (bottom right) (*p* = non-significant, *n* = 12). Statistical comparisons were performed by the log-rank test

A peritoneal disseminated tumour model was established using the mouse ovarian cancer cell lines HM-1-VISTA and HM-1-control. Spleen cells and peritoneal tumours were harvested and the percentages of T cells, myeloid-derived suppressor cells (MDSCs), and IFN-γ-producing cells were measured by flow cytometry. VISTA expression significantly decreased the number of CD8+ T cells at the tumour site but had no obvious difference on the number of CD4+ T cells (Fig. 5a). There was no difference in the number of CD8+ and CD4+ T cells in the spleen (Supplementary Figure 8C). Interestingly, the percentage of IFN-γ-producing cells at the tumour site was reduced when VISTA was overexpressed (Fig. 5a). Moreover, more MDSCs were detected in spleens and tumour sites of mice transplanted with VISTA-overexpressing cells than in mice transplanted with control cells (Supplementary Figure 8D).

To further assess the effect of VISTA in tumour cells on survival, we intraperitoneally injected HM-1-VISTA or HM-1-control cells along with ID8-VISTA or ID8-control cells into immunocompetent or immunodeficient mice. No survival difference was detected in immunodeficient mice, but in immunocompetent mice transplanted with cells overexpressing VISTA, survival was significantly shortened (Fig. 5b). These results demonstrated that VISTA expression in the tumour induces immunosuppression in host immune cells, leading to a poor prognosis. Collectively, these data suggested that VISTA in tumour cells negatively regulates antitumour immunity in ovarian cancer.

### Blocking of VISTA in tumour cells extends survival of mice inoculated with ovarian cancer cells overexpressing VISTA

**Fig. 6:**
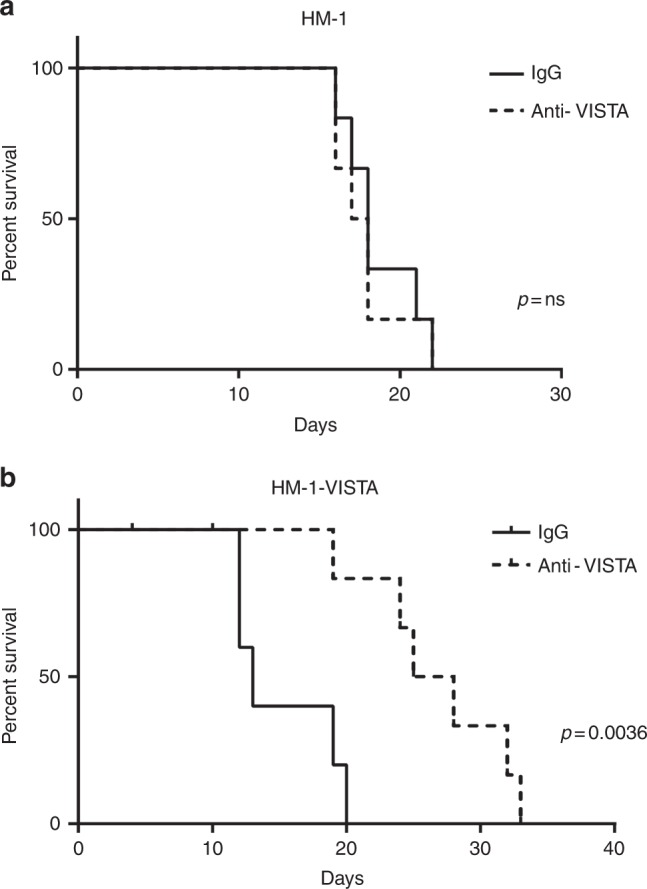
Anti-VISTA antibody treatment in a high VISTA-expressing tumour model. **a** Survival curves for mice intraperitoneally inoculated with HM-1 mouse ovarian cancer cells treated with anti-VISTA antibody or IgG (*p* = non-significant, *n* = 6; log-rank test). **b** VISTA-overexpressing HM-1 cells were intraperitoneally injected into syngeneic mice, which were then treated with anti-VISTA antibody or IgG (*p* = 0.0036, *n* = 6; log-rank test)

Given the ability of VISTA to inhibit T cell responses, we investigated the benefit of blocking VISTA. To this end, we first performed anti-VISTA antibody (MIH63) therapy on HM-1 injected mice but observed no improvement in survival (Fig. 6a). Next, we conducted the same treatment in mice injected with HM-1-VISTA. Mice treated with anti-VISTA antibody exhibited significantly prolonged survival in comparison with the control group (Fig. 6b).

One of the challenges confronting current PD-1 blockade immunotherapy is that some patients are resistant to this therapy, and no strategy to overcome this resistance has yet been established. We performed anti-PD-1 therapy or a combination of anti-PD-1 with anti-VISTA therapy. Interestingly, anti-PD-1 antibody therapy did not improve the survival rate of HM-1-VISTA-injected mice relative to controls, suggesting that HM-1-VISTA exhibits primary resistance to PD-1 blocking therapy. Since PD-1 and VISTA function independently,^31,32^ we next explored the efficacy of an anti-VISTA antibody. Single-agent anti-VISTA treatment significantly prolonged survival, but the combination of anti-VISTA with anti-PD-1 did not improve survival relative to single-agent anti-VISTA treatment (Supplementary Figure 8E). These results suggest that anti-VISTA therapy may be effective for anti-PD-1 therapy-resistant ovarian cancers that express VISTA.

## Discussion

VISTA is a novel immune-checkpoint molecule whose expression has been mainly reported in hematopoietic and myeloid cells, and to a lesser extent in T cells. VISTA is critical in regulating immunosuppression.^24,35–38^ Its expression in tumour cells has recently been studied.^39^ However, no report to date has described the function of VISTA in tumour cells. In this study, we conducted a comprehensive characterisation of VISTA transcript and protein expression in cancer cells. We found that clinical samples with high VISTA expression in tumour cells exhibited characteristics of immunosuppression.

Both ovarian and endometrial cancers more frequently expressed VISTA in the tumour cells than in normal cells. IHC analysis of VISTA with polyclonal antibody revealed staining of both the cytoplasm and membrane.

VISTA is a type I transmembrane protein that contains an IgV domain in its extracellular region. The extracellular domain of VISTA is linked to a stalk region, transmembrane segment, and a cytoplasmic domain.^24^ The staining pattern was determined by the location of VISTA staining, since the polyclonal antibody targeted both extracellular and cytoplasmic domains, similar to the demonstration of PD-L1 expression.^40–42^ The cytoplasmic staining may represent intracellular stores of VISTA that subsequently are expressed on the cell surface given the appropriate stimulation.^43^ Further studies are needed to verify this speculation. In addition, VISTA expression does not correlate with patient outcome in clinical settings, probably due to the complex interaction between multiple immune-checkpoint molecules and the weak suppressive function of VISTA in tumour cells.

Immunofluorescence of endometrial cancer cells with double staining of VISTA and CD8 revealed the presence of VISTA-positive CD8+ T cells in two of four patient samples. The finding may indicate that VISTA expression in CD8+ T cells might be related to immune cell infiltration at the tumour site, while mechanism of this was unknown (Supplementary Figure 1J).

Using the established VISTA-expressing ID8 and HM-1 mouse ovarian cell lines, we showed that antigen-specific cytolysis by CTLs was inhibited by VISTA expression in tumour cells, and that VISTA significantly inhibited T cell proliferation. Consistent with these results, silencing of VISTA in both human endometrial and ovarian cancer cells reversed this inhibition. In mice harbouring ovarian cancer cells expressing high levels of VISTA, we observed a significant decrease in the abundance of CD8+ T cells, infiltration by IFN-γ-producing T cells, and upregulation of MDSC accumulation in the tumour microenvironment. Such dynamic immunological alterations within the tumour microenvironment reflect the accelerated migration of MDSCs into tumour sites or increased infiltration of immature myeloid cells, which might differentiate into MDSCs within the tumour site.^44,45^ However, the mechanisms underlying the effect of VISTA on MDSCs remain unknown. Thus, our results indicate that beyond promoting cancer progression by engaging with T cells that express VISTA, tumour cells that express VISTA may also promote tumour immune evasion.

The mechanisms underlying VISTA expression in cancer cells are unclear. A few reports have described the relationship between miRNAs (miR-125 and miR-506) and VISTA expression.^33,46^ However, we did not detect evidence of a correlation between the levels of these miRNAs and VISTA expression in several ovarian and endometrial cancer cell lines. Treatment with various cytokines, including IFN-γ, IL-2, IL-4, and IL-6, did not increase VISTA expression in tumour cells. On the other hand, decitabine treatment induced VISTA upregulation in several endometrial cancer cell lines, but not in ovarian cancer cell lines. These results suggest that VISTA expression in endometrial cancer might be associated with promoter methylation status. To test this hypothesis, we sequenced several *VISTA* promoter regions in high or low VISTA-expressing endometrial cancer cell lines and clinical samples. The results revealed that hypomethylation of the *VISTA* promoter region leads to high VISTA expression in endometrial cancers. Thus, VISTA expression in cancer cells might indeed be regulated by promoter methylation status. Similar results were not obtained in ovarian cancers, suggesting that expression is regulated in a cell type-specific manner.

In the mouse model of anti-VISTA antibody treatment, it should be noted that the antibody blocked both VISTA expressed on tumour cells and immune cells. However, a significant survival benefit was observed in VISTA-overexpressing tumours compared to the control group, indicating the stronger inhibitory effect of VISTA for tumour side than on immune cells. Several dual immunotherapy combination regimens are currently under exploration. A recent study reported that VISTA expression is upregulated in myeloid cells after treatment with anti-CTLA-4 antibody (ipilimumab) and might promote resistance to CTLA-4 blockade.^47^ Although it remains unknown whether anti-PD-1 antibody treatment also upregulates VISTA expression in the tumour microenvironment, resistance to anti-PD-1 therapy is a major problem that must be solved to advance to the next stage of cancer immunotherapy. Previous studies suggested that immune-checkpoint molecules, such as PD-1, CTLA-4, and VISTA, non-redundantly regulate antitumour responses.^31,32^ Our preclinical results showed that in the presence of immune-suppressive signalling PD-1, blocking of VISTA led to immune reactivation in the mouse HM-1-VISTA model. Therefore, blocking of VISTA may induce antitumour activity in the tumour microenvironment, even when PD-1 is protecting tumour cells from immunosurveillance. Thus, anti-VISTA antibody therapy represents a potential treatment option for VISTA-expressing tumours that are resistant to anti-PD-1 therapy.

In conclusion, the VISTA-mediated immune inhibitory pathway in tumour cells regulates protective antitumour immunity, and blocking of VISTA in tumour cells may provide a promising immunotherapeutic strategy for improving the antitumour response.

## Electronic supplementary material

Supplementary figure legends

Supplementary figure 1

Supplementary figure 2

Supplementary figure 3

Supplementary figure 4

Supplementary figure 5

Supplementary figure 6

Supplementary figure 7

Supplementary figure 8

## Availability of data and material

The data that support the findings of this study are available from the corresponding author upon reasonable request.
