# Supplementary material for: VISTA expressed in tumour cells regulates T cell function
Source: Br J Cancer. 2018 Nov 9;120(1):115–27. doi: 10.1038/s41416-018-0313-5 (PMC6325144; doi:10.1038/s41416-018-0313-5)
Supplement: Supplementary file 1 — Supplementary figure legends [file 41416_2018_313_MOESM1_ESM.docx]

**Supplementary Figure Legends**

**Supplementary Figure 1. Expression of VISTA in gynecologic tumors**

(**A**) RNA expression of VISTA in 30 types of malignant tumors in the The Cancer Genome Atlas database. Abbreviations are: ACC, adrenocortical carcinoma; ALL, acute myeloid leukemia; DLBC, diffuse large B cell lymphoma; GBM, glioblastoma multiform; PCPG, pheochromocytoma and paraganglioma; ccRCC, clear cell renal cell carcinoma; chRCC, chromophobe renal cell carcinoma; and pRCC, papillary renal cell carcinoma. Each dot represents one case in the dataset. (**B**) VISTA gene expression in endometrial cancer tissue vs. normal endometrioid tissue in the GSE17025 microarray dataset. A significant difference was evident (p = 0.0361) using the Mann–Whitney *U* test. (**C**) VISTA immunohistochemistry (IHC) score in different stages of endometrial cancer. One-way ANOVA multiple comparisons were used. (**D**) Survival of endometrial cancer patients expressing high levels of VISTA vs. survival of patients expressing low levels of VISTA. The difference was non-significant difference by the log–rank test. (**E**) VISTA IHC score in 28 primary tumors and corresponding metastasis specimens. The difference was non-significant (p = 0.1127) by the paired t test. (**F**) VISTA IHC score in different stages of ovarian cancer assessed by one-way ANOVA multiple comparisons. (**G**) Survival curve of ovarian cancer patients expressing high levels of VISTA vs. survival of patients expressing low levels of VISTA. The difference was non-significant by the log–rank test. (**H**) Representative IHC staining of three endometrial cancer specimens and three ovarian cancer specimens using polyclonal antibody or two different monoclonal antibodies. (**I**) Correlation of IHC score obtained with the polyclonal antibody with those obtained with each monoclonal antibody. Monoclonal antibody 1: n= 10, Relative Risk (RR) =0.7203, p=0.0019. Monoclonal antibody 2: n=10, RR=0.5427, p=0.0151. (**J**) Representative immunofluorescence staining of two endometrial cancer specimens using anti-VISTA antibody (red), anti-CD8 antibody (green), and nuclear antibody (red). Left panel displays a representative case of enhanced VISTA-positive CD8+T cells and the right panel displays a representative case of poor VISTA-positive CD8+T cells. Scale bar denotes 100 μm.

**Supplementary Figure 2. Expression of VISTA in cancer cell lines and established sh-VISTA cell lines**

(**A**) VISTA expression was determined by RT-PCR in 12 endometrial cancer cell lines and one immortalized endometrial epithelial cell line (EM). (**B**) VISTA expression was determined by RT-PCR in nine ovarian cancer cell lines. (**C**) VISTA expression was determined by western blotting in nine endometrial cancer cell lines and one immortalized endometrial epithelial cell line (EM). (**D**) VISTA expression was determined by western blotting after treatment of human endometrial cancer cell lines or immortalized endometrial epithelial cell line (EM) with 20 ng/ml IFN-γ or 5 ng/ml TGFβ. (**E**) Generation of the three independent human endometrial cancer HEC1A sh-VISTA cell lines (left) and human ovarian cancer COV504 sh-VISTA cell lines (right). VISTA silencing was confirmed by RT-PCR and western blotting.

**Supplementary Figure 3.** **Silencing of VISTA expression in human ovarian/ endometrial cancer cells restores T-cell proliferation**

T cells were purified from human PBMCs by magnetic bead selection. Cells were labeled with carboxyfluorescein succinimidyl ester (CFSE), stimulated with CD3/CD28 beads, and co-cultured with mitomycin-treated sh-control or sh-VISTA endometrial **(A)** or ovarian **(B)** tumor cells at a 1:1 ratio. T-cell proliferation was assessed using CFSE. Data are representative of three separate experiments. Histogram of flow cytometry data is displayed in the upper panel and percentage of each generation is displayed in the lower panel.

**Supplementary Figure 4.** **miR-125a and miR-506 mRNA expression in cancer**

Levels of miR-125a (**A**) and miR-506 (**B**) were determined by RT-PCR. No correlation was detected between the levels of these miRNAs and *VISTA* mRNA levels in human endometrial cancer or ovarian cancer.

**Supplementary Figure 5.** **Methylation status of promoter region 2 in human ovarian cancer**

(**A**) VISTA expression in ovarian cancer cell lines after decitabine treatment was measured using RT-PCR. Data are presented as mean ± SEM (n = 6). (**B**) Positional relationship of the three selected regions of the *VISTA* promoter. Scale bar denotes 100 bp. (**C**) Methylation status of promoter region 2 in clinical ovarian cancer specimens expressing high or low levels of VISTA.

**Supplementary Figure 6. Methylation status of promoter region 1 in endometrial cancer**

(**A**) Positional relationship of the three selected regions of the *VISTA* promoter. Scale bar denotes 100 bp. (**B**) Methylation status of promoter Region 1 in the high VISTA-expressing JHUEM1endometrial cancer cell line. (**C**) Methylation status of promoter region 1 in the low VISTA-expressing JHUEM7 endometrial cancer cell line. Solid circle indicates the methylated site and hollow circle indicates the unmethylated site. Each column represents a CpG site and each horizontal row indicates a different sample.

**Supplementary Figure 7.** **Methylation status of promoter region 3 in endometrial cancer and methylation status of promoter Region 2 in an immortalized endometrial epithelial cell line**

(**A**) Positional relationship of the three selected promoter regions of *VISTA*. Scale bar denotes 100 bp. (**B**) Methylation status of promoter region 3 in the high VISTA-expressing JHUEM1 endometrial cancer cell line. (**C**) Methylation status of promoter region 3 in the low VISTA-expressing JHUEM7endometrial cancer cell line. (**D**) Positional relationship of the three selected regions of the *VISTA* promoter. Scale bar denotes 100 bp. (**E**) The immortalized endometrial epithelial cell line (EM) exhibited a moderate level of methylation in promoter Region 2.

**Supplementary Figure 8. T-cell or MDSC distribution after injection with a VISTA-overexpressing tumor cell line and anti-PD-1 therapy on VISTA overexpressing tumor**

**(**A) VISTA expression in HM-1 and ID8 mouse ovarian cancer cell lines after exposure to various cytokines. Filled gray indicates the isotype control and the dashed line indicates VISTA staining. Cytokines were added to the medium 24 hours before the assessment. Data are from one representative experiment of the three repeat experiments that yielded similar results. (**B**) Proliferation of VISTA-overexpressing HM-1 and ID8 cells. Data are from one representative experiment of three repeat experiments that yielded similar results. (**C**) Spleen and peritoneal tumors were harvested from mice injected with HM-1-VISTA or control cells, and T cell distribution was evaluated by flow cytometry. VISTA had no effect on the distribution of CD4+ T cells, CD8+ T cells, or IFN-γ–producing cells in the spleen. Data are shown as mean ± SEM (n = 6, Mann Whitney *U* test). (**D**) VISTA upregulates myeloid-derived suppressor cell (MDSC) accumulation in spleen and tumor. Data are shown as mean ±SEM (n = 6, **p < 0.01 by Mann–Whitney *U* test). (**E**) Anti-VISTA antibody (MIH63) or a combination of anti-VISTA antibody and anti-PD-1 antibody were used to treat HM-1-VISTA tumor model mice. Anti-VISTA vs. IgG, p < 0.0001, n = 6; anti- PD-1 vs. IgG, p=ns, n = 6. Anti-VISTA vs. the combination of anti-VISTA and anti-PD-1 antibody (p = ns, n = 6, log-rank test).
