# Supplementary figures and images for: VISTA expressed in tumour cells regulates T cell function
Source: Br J Cancer. 2018 Nov 9;120(1):115–27. doi: 10.1038/s41416-018-0313-5 (PMC6325144; doi:10.1038/s41416-018-0313-5)

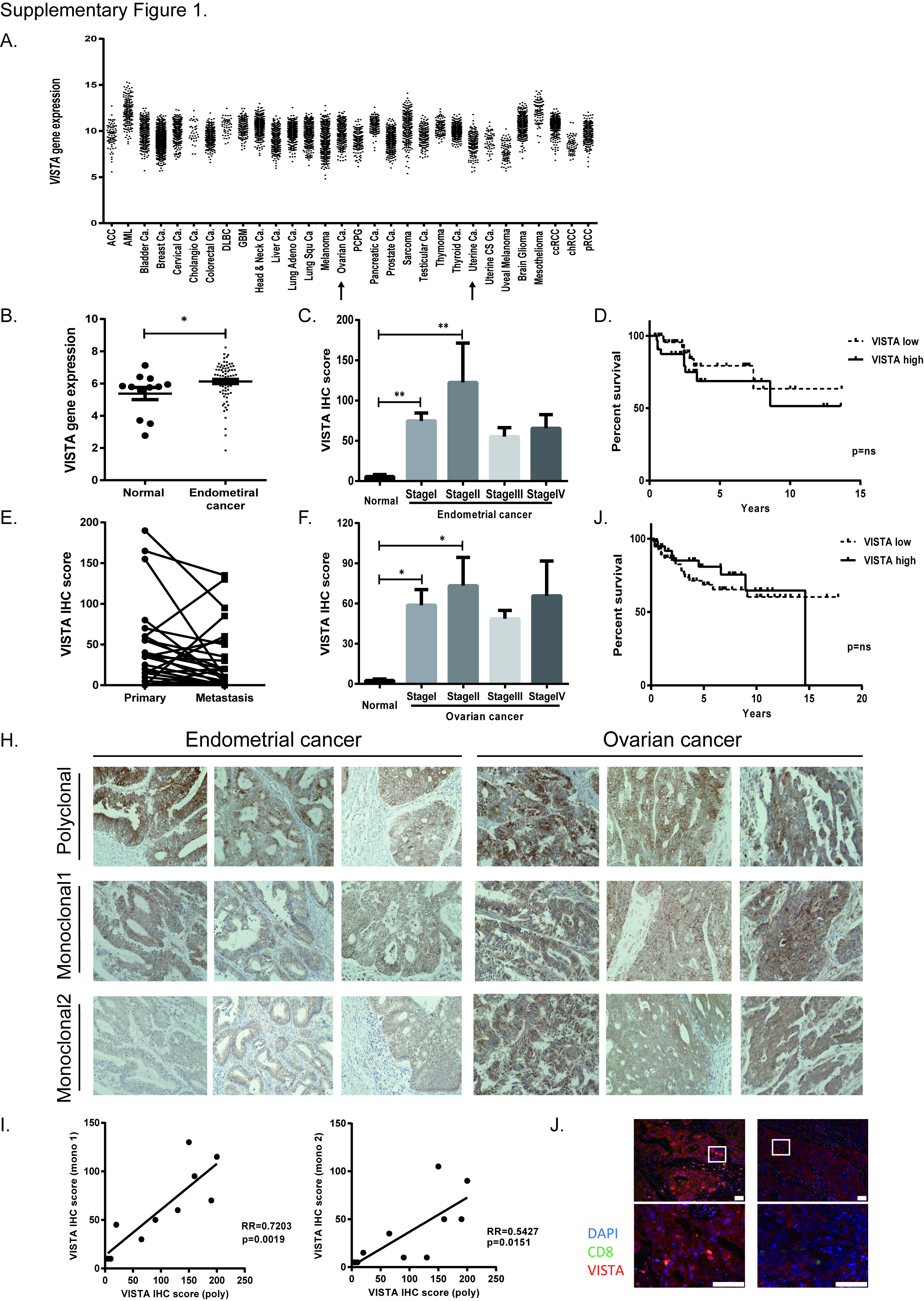

Supplement: Supplementary file 2 — Supplementary figure 1 [file 41416_2018_313_MOESM2_ESM.tif]

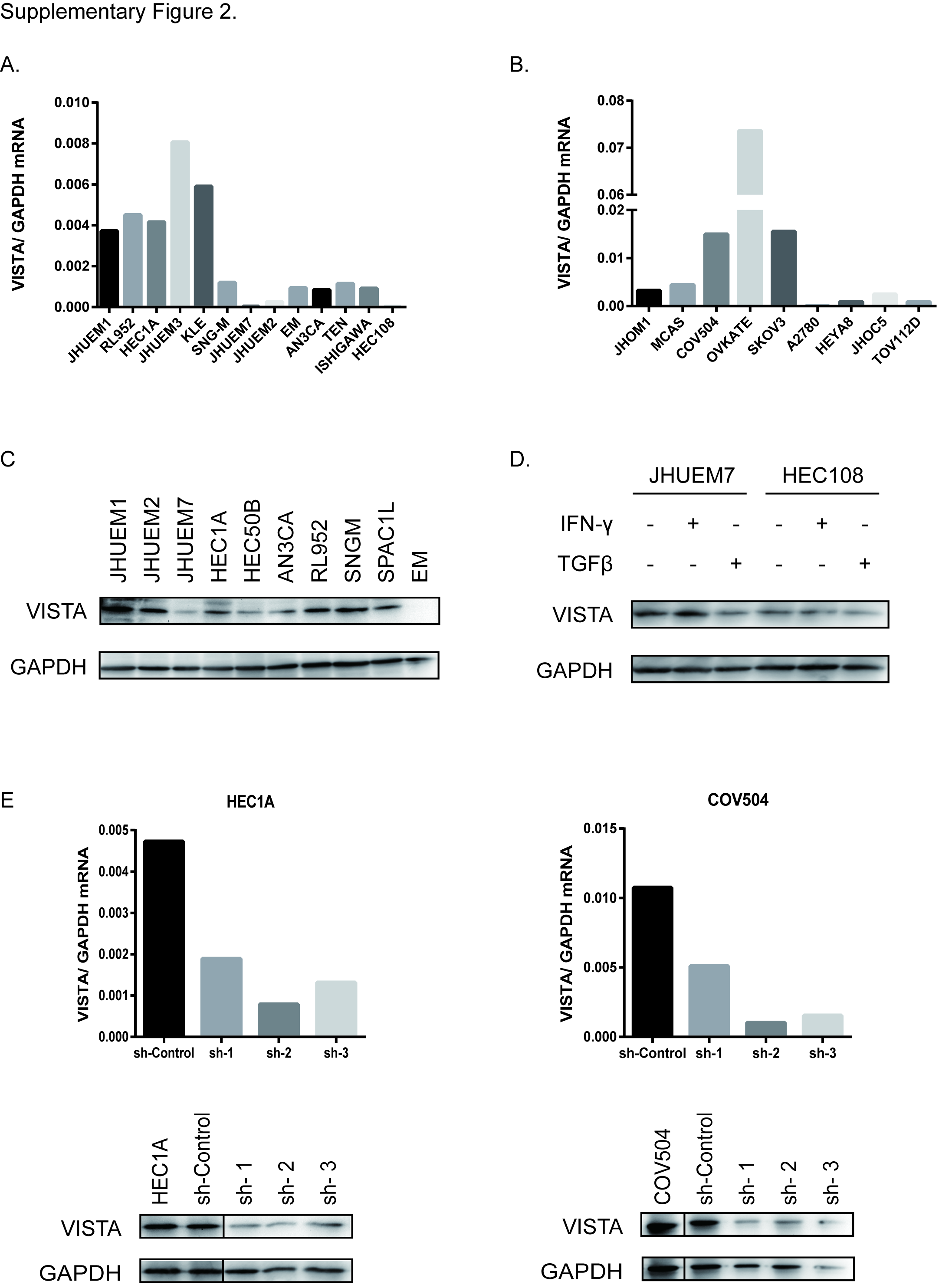

Supplement: Supplementary file 3 — Supplementary figure 2 [file 41416_2018_313_MOESM3_ESM.tif]

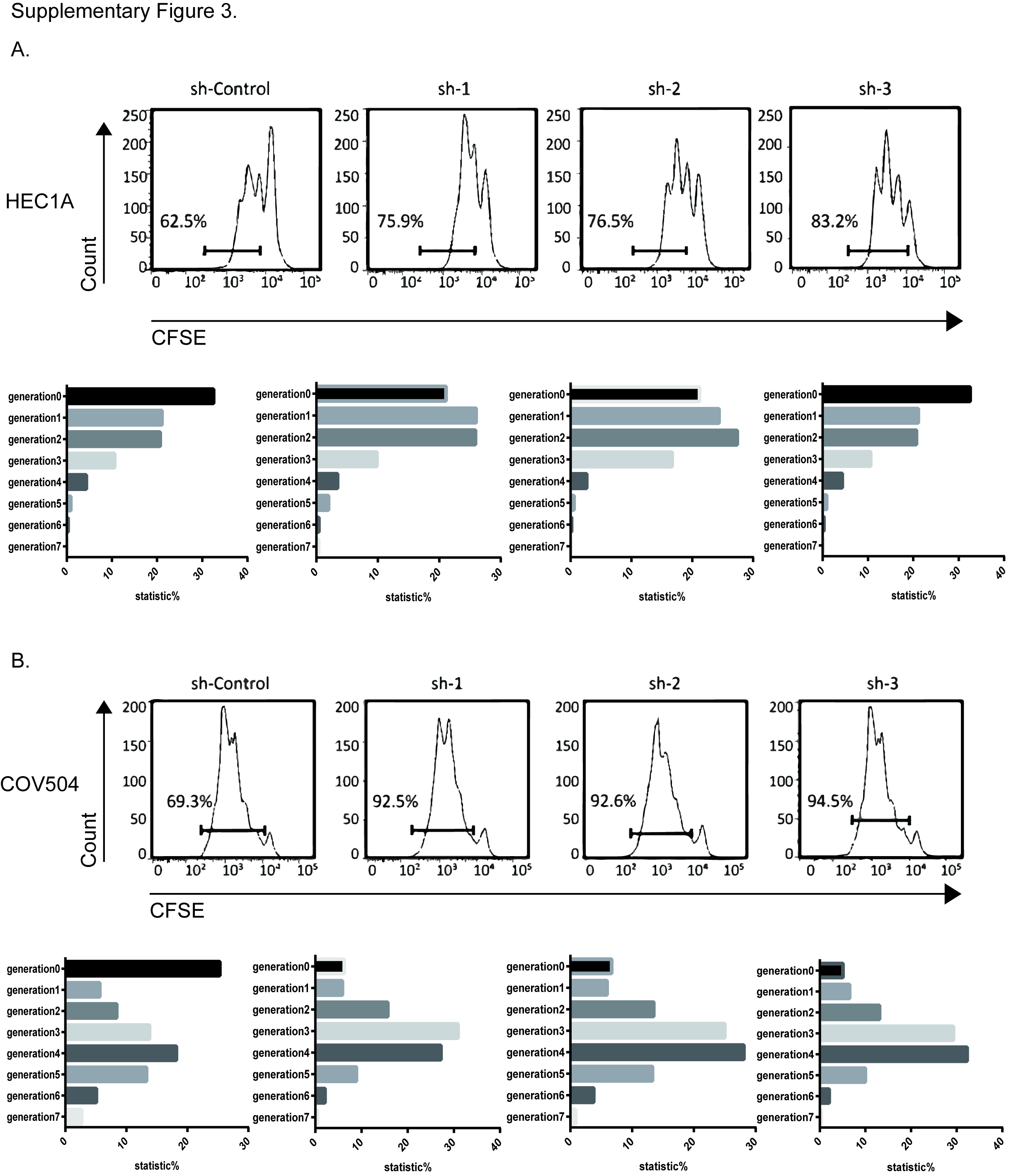

Supplement: Supplementary file 4 — Supplementary figure 3 [file 41416_2018_313_MOESM4_ESM.tif]

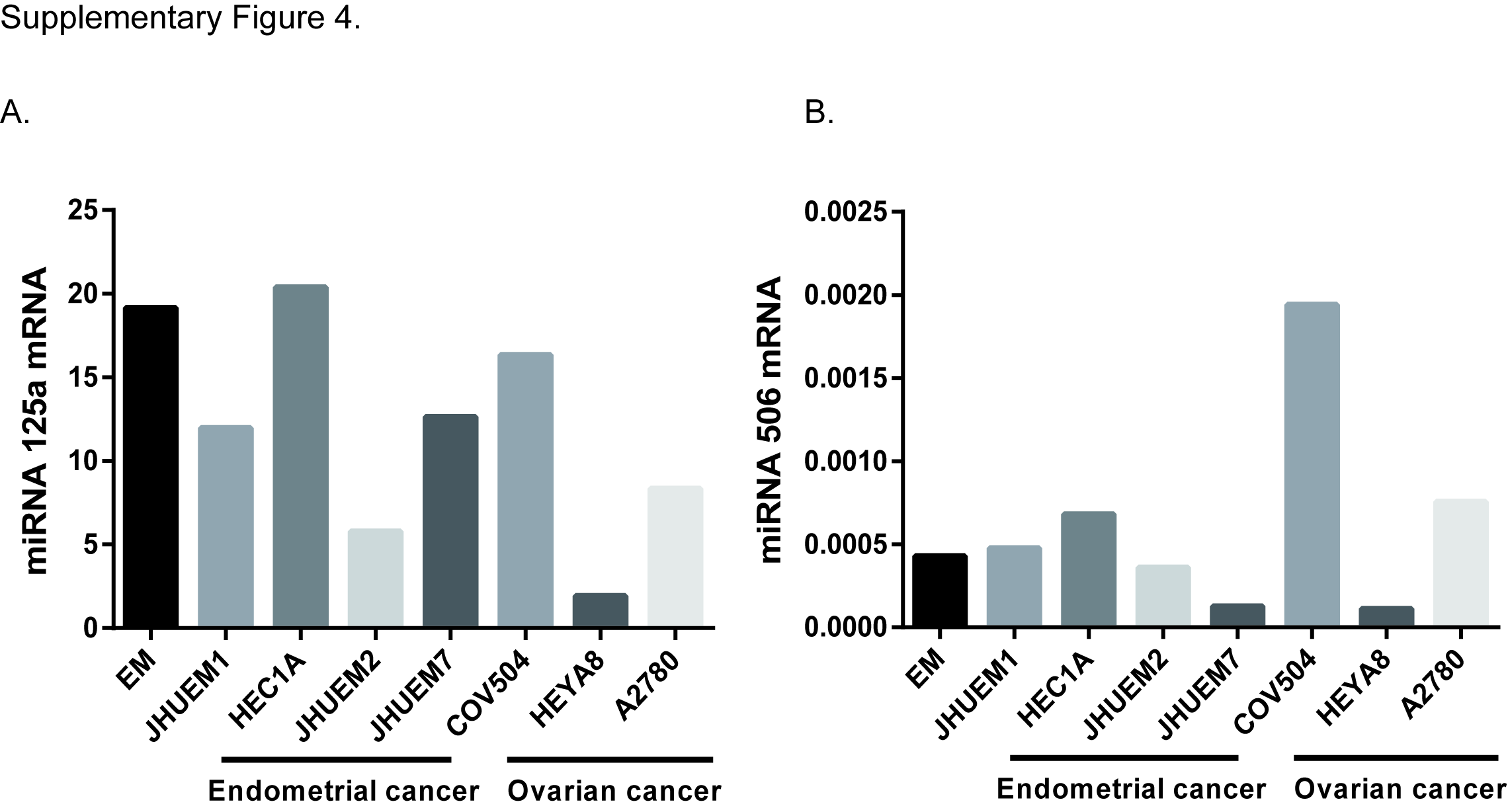

Supplement: Supplementary file 5 — Supplementary figure 4 [file 41416_2018_313_MOESM5_ESM.tif]

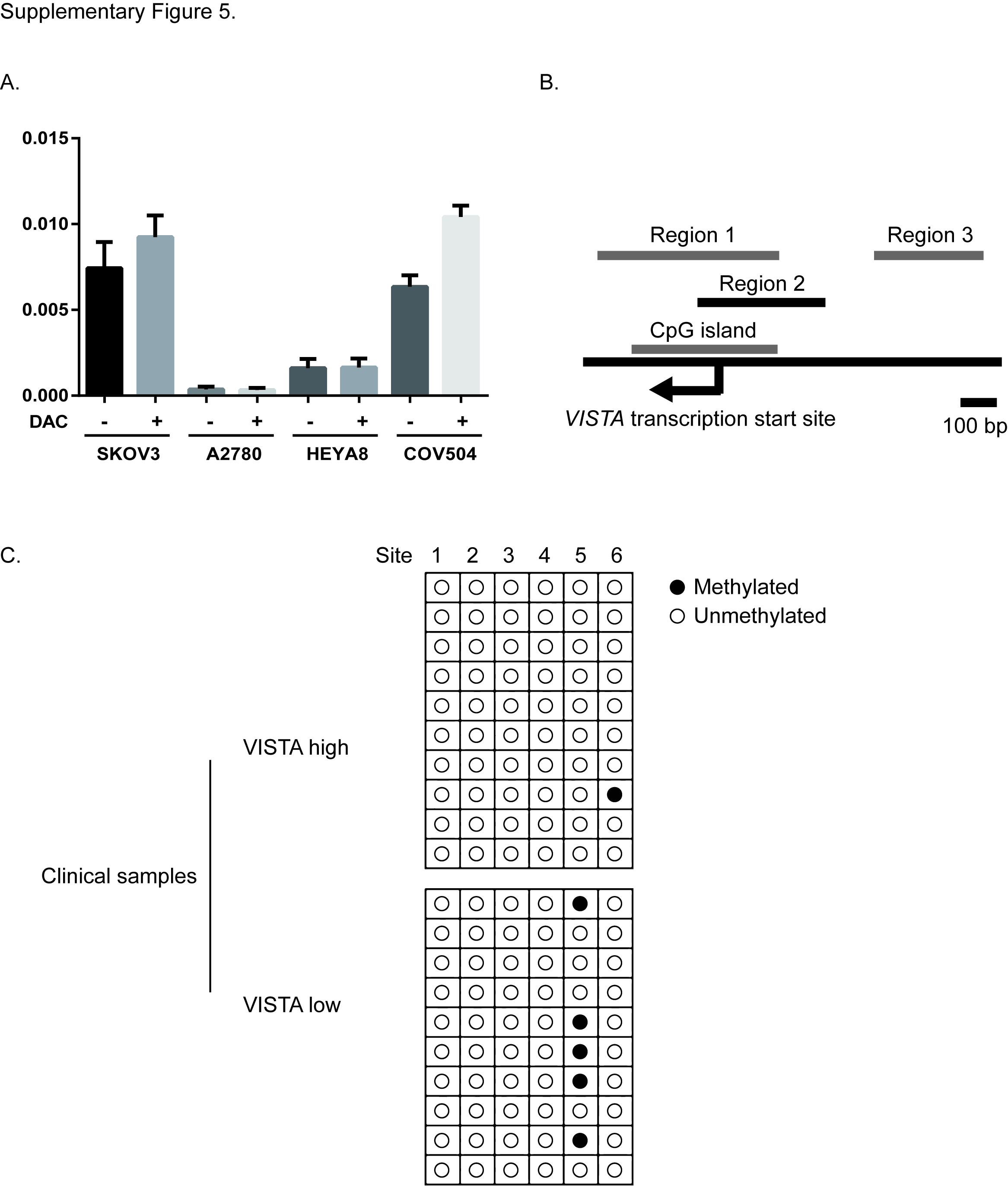

Supplement: Supplementary file 6 — Supplementary figure 5 [file 41416_2018_313_MOESM6_ESM.tif]

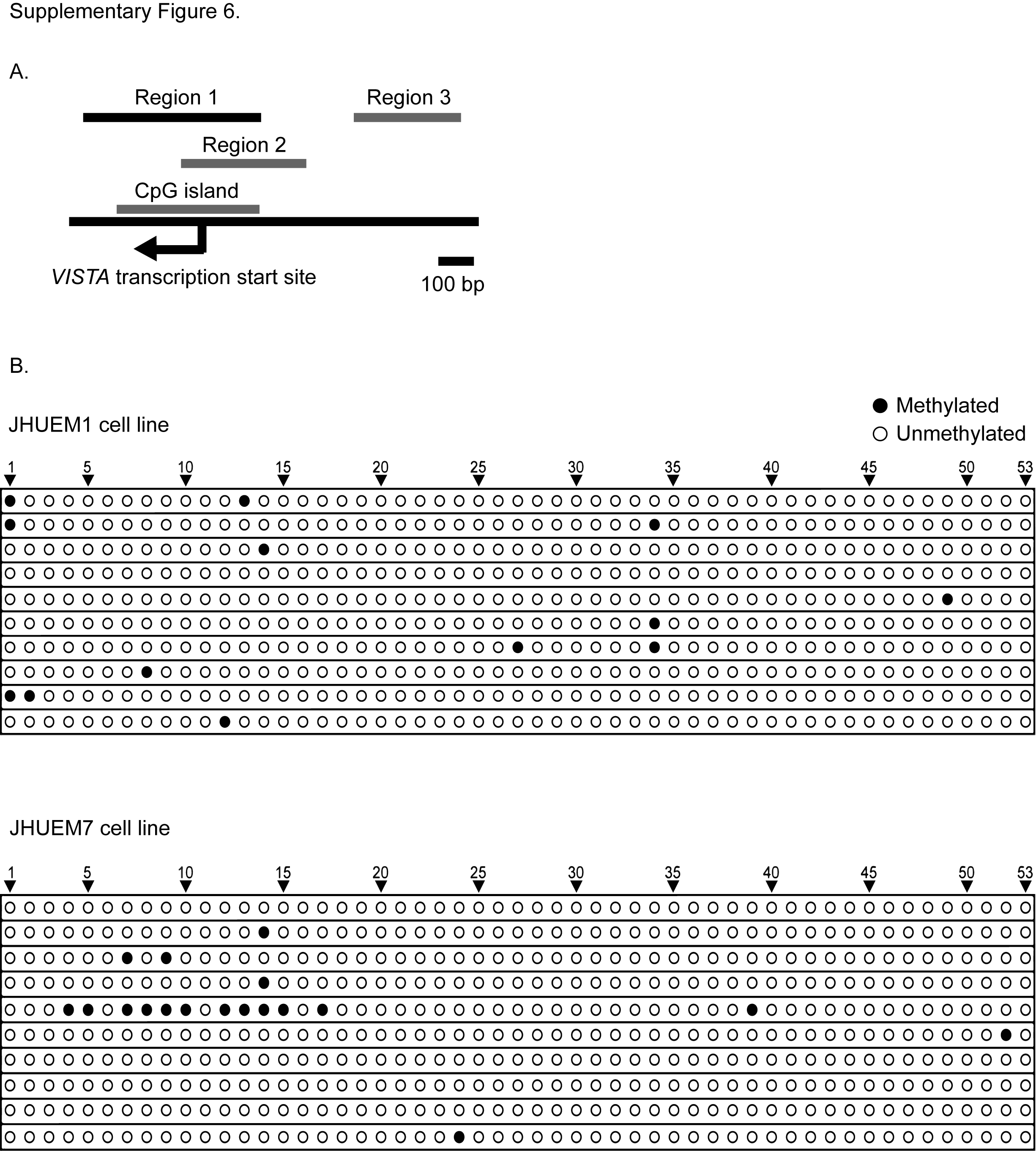

Supplement: Supplementary file 7 — Supplementary figure 6 [file 41416_2018_313_MOESM7_ESM.tif]

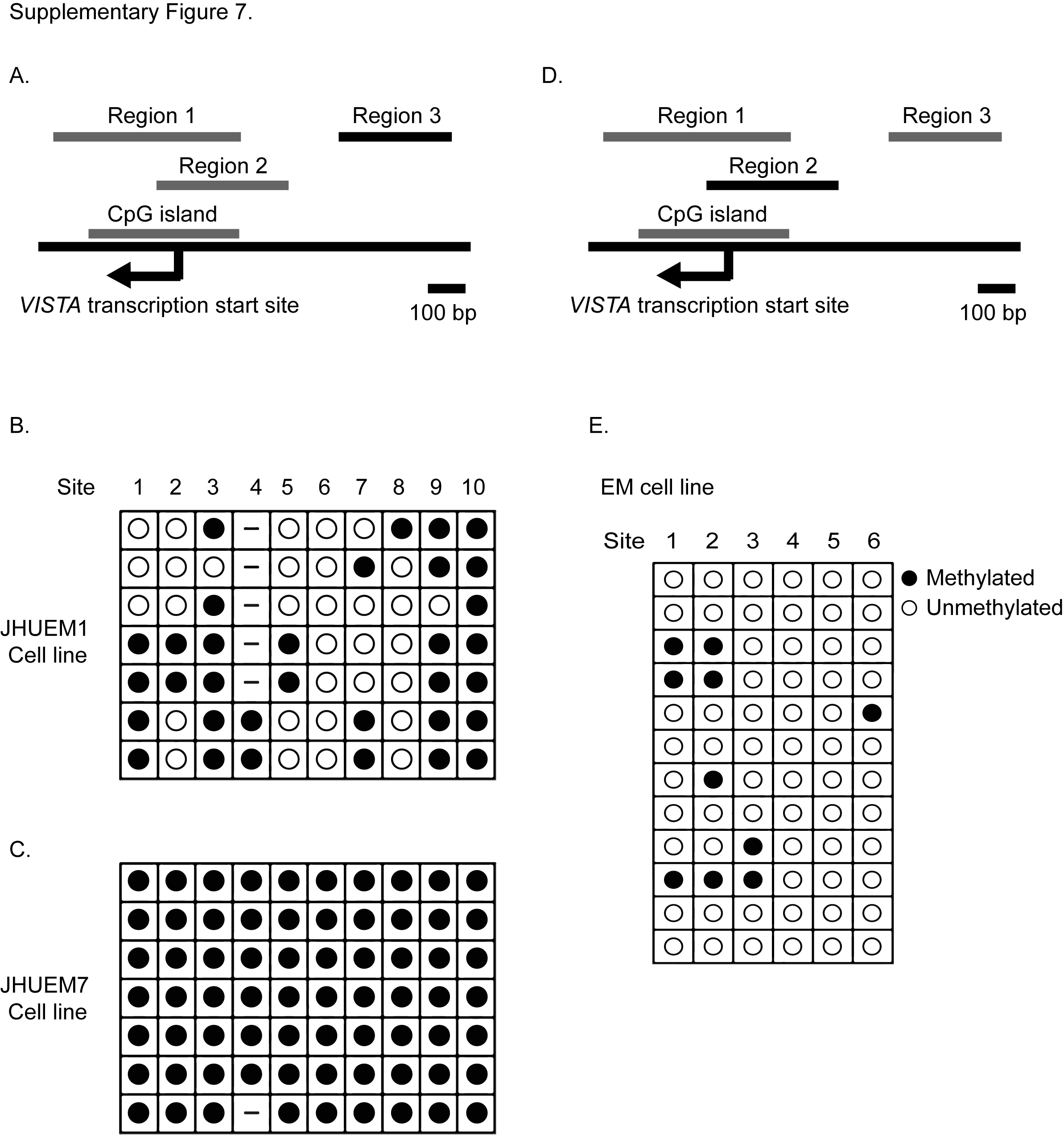

Supplement: Supplementary file 8 — Supplementary figure 7 [file 41416_2018_313_MOESM8_ESM.tif]

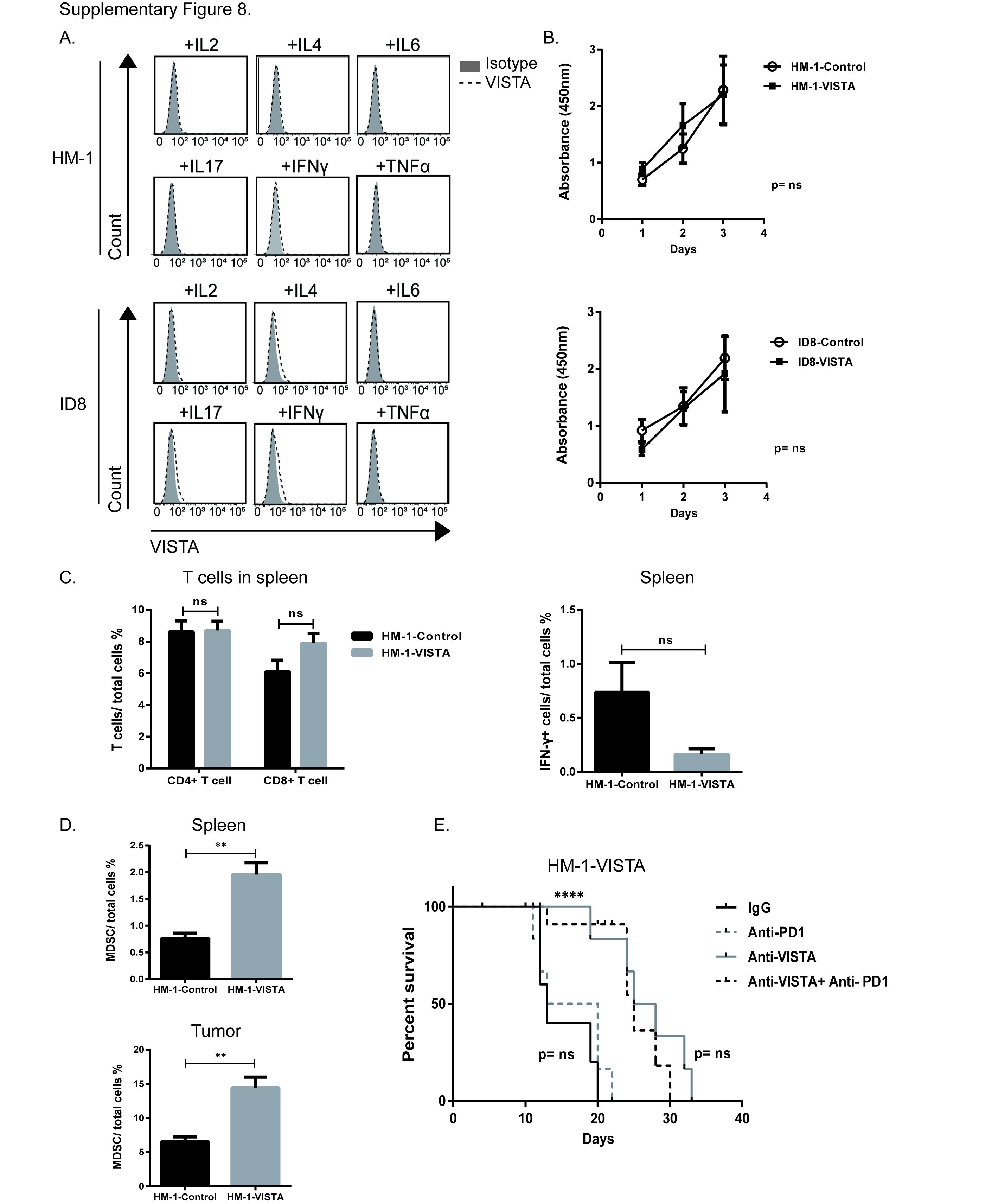

Supplement: Supplementary file 9 — Supplementary figure 8 [file 41416_2018_313_MOESM9_ESM.tif]
